# Effects of follicle‐stimulating hormone followed by gonadotropin‐releasing hormone on embryo production by ovum pick‐up and in vitro fertilization in the river buffalo (*Bubalus bubalis*)

**DOI:** 10.1111/asj.13196

**Published:** 2019-03-10

**Authors:** Kenichiro Sakaguchi, Excel Rio S. Maylem, Ramesh C. Tilwani, Yojiro Yanagawa, Seiji Katagiri, Edwin C. Atabay, Eufrocina P. Atabay, Masashi Nagano

**Affiliations:** ^1^ Laboratory of Theriogenology Graduate School of Veterinary Medicine Hokkaido University Sapporo Japan; ^2^ Research Fellow of Japan Society for the Promotion of Science Tokyo Japan; ^3^ Reproductive Biotechnology and Physiology Laboratory Philippine Carabao Center National Headquarters Science City of Munoz, Nueva Ecija Philippines; ^4^ Laboratory of Theriogenology Department of Clinical Sciences Faculty of Veterinary Medicine Hokkaido University Sapporo Japan

**Keywords:** FSH, GnRH, OPU‐IVF, Water buffalo

## Abstract

In this study, we examined the effects of superstimulation using follicle‐stimulating hormone (FSH) followed by gonadotropin‐releasing hormone (GnRH) on buffalo embryo production by ultrasound‐guided ovum pick‐up (OPU) and in vitro fertilization (IVF). Nine Murrah buffaloes were subjected to OPU‐IVF without superstimulation (control). The morphologies of the oocytes collected were evaluated, and oocytes were then submitted to in vitro maturation (IVM). Two days after OPU, same nine buffaloes were treated with twice‐daily injections of FSH for 3 days for superstimulation followed by a GnRH injection. Oocytes were collected by OPU 23–24 hr after the GnRH injection and submitted to IVM (the superstimulated group). The total number of follicles, number of follicles with a diameter > 8 mm, and number of oocytes surrounded by multi‐layered cumulus cells were higher in the superstimulated group than in the control group (*p* ≤ 0.05). After IVF, the percentages of cleavage and development to blastocysts were higher in the superstimulated group than in the control group (*p* < 0.05). In conclusion, superstimulation improved the quality of oocytes and the embryo productivity of OPU‐IVF in river buffaloes.

## 1 INTRODUCTION

Water buffaloes are important livestock in developing countries in southeastern Asia, and have contributed to the local economy (Mishra et al., 2015; Colli et al., 2018). There are two types of water buffaloes, the swamp buffalo (*Bubalus carabanensis*) and river buffalo (*Bubalus bubalis*). The swamp buffalo has been used for draft power in a wide area ranging from eastern India (the Assam region) through southeastern Asia, and Indonesia to eastern China (Zhang et al., 2016), while the river buffalo has been improved as a dairy breed and spreads from the Indian subcontinent to eastern Mediterranean countries (Colli et al., 2018). To increase milk production, river buffaloes have been imported to eastern Asia, southern America, and central Africa (Kierstein et al., 2004). Three thousand Murrah buffaloes, one breed of river buffalo, were imported from Bulgaria to the Philippines to increase milk production by the native swamp buffalo, called Carabao, through cross breeding (Borghese, 2011). Although the cross breeding of Carabao (milk production: 400 kg per lactation) and Murrah buffaloes (1,800 kg per lactation) resulted in increased milk production by the crossbred buffaloes (1,100 kg per lactation) (Borghese, 2011), reproduction was compromised by heteroploidy due to differences in chromosome numbers (swamp buffalo: 2n = 48, river buffalo: 2n = 50). Therefore, a strategy to maintain and effective increasing of the genetic resources in pure river buffaloes has become important for increasing milk production. Although the superovulatory treatment and embryo transfer technique using in vivo‐derived embryos are effective for achieving genetic improvements in cattle (Hasler, 2014), buffaloes respond poorly to superstimulation and embryo recovery is globally lower than that in cattle; total and transferable embryos of 10.0 and 6.7, respectively, in cattle (Perry, 2017), but only 2.3–2.8 and 1.4–1.6, respectively, in buffaloes (Mishra, 1997; Neglia et al., 2010; Li et al., 2011).

Ultrasound‐guided ovum pick‐up (OPU) combined with the in vitro fertilization (IVF) technique is now widely used to produce embryos in cattle for genetic improvements (Hasler, 2014) because the efficiency of embryo production by OPU‐IVF is higher than that by in vivo embryo production concomitant with the superovulatory treatment (Pontes et al., 2009). This technology has been also applied to buffalo species (Galli, Duchi, Colleoni, Lagutina, & Lazzari, 2014). However, the average number of blastocysts per session of OPU‐IVF was previously reported to be fewer in buffalo species than in cattle (buffaloes vs. cattle; 1.07 vs. 2.49 embryos, respectively) (Galli et al., 2014). On the other hand, the production rate of transferable embryos after OPU‐IVF was similar in both species (buffaloes vs. cattle; 16.2% and 19.4%, respectively) (Galli et al., 2014). The lower productivity of embryos in buffaloes has been attributed to the smaller number of follicles developed during the estrous cycle (Manik, Palta, Singla, & Sharma, 2002). Superstimulation using follicle‐stimulating hormone (FSH) increases the number of follicles for OPU and embryo productivity in cattle (De Roover, Genicot, Leonard, Bols, & Dessy, 2005; Hasler, 2014). Moreover, superstimulation followed by a gonadotropin‐releasing hormone (GnRH) treatment was previously shown to induce a luteinizing hormone (LH) surge, producing in vivo‐matured oocytes, which had higher developmental competence than in vitro‐matured oocytes, in cattle (Matoba et al., 2014). Previous studies attempted FSH treatments followed by GnRH or human chorionic gonadotropin (hCG) for OPU‐IVF in buffaloes (Presicce et al., 2002; Techakumphu, Promdireg, Na‐Chiengmai, & Phutikanit, 2004; Promdireg, Adulyanubap, Singlor, Na‐Chiengmai, & Techakumphu, 2005). These studies only focused on the morphologies of the oocytes collected, and did not investigate the developmental competence of oocytes after FSH and GnRH or hCG injections. In this study, we investigated the effects of a FSH treatment followed by a GnRH injection on follicular development, oocyte quality, and the embryo productivity by OPU‐IVF in river buffaloes.

## 2 MATERIALS AND METHODS

### 2.1 Animals

Nine Murrah buffaloes maintained at the Philippine Carabao Center in Science City of Muñoz were used in this study. They were provided with daily rations of concentrates, fresh forage, and rice straw. Their age and parity were 8.2 ± 3.9 years and 2.3 ± 2.4, respectively (mean ± standard deviation). This study was approved by the Institutional Animal Care and Use Committee of the Philippine Carabao Center.

### 2.2 Chemicals

All chemicals used in this study were purchased from Sigma‐Aldrich (St. Louis, MO, USA) unless otherwise stated.

### 2.3 OPU for in vitro embryo production

**Figure 1:**
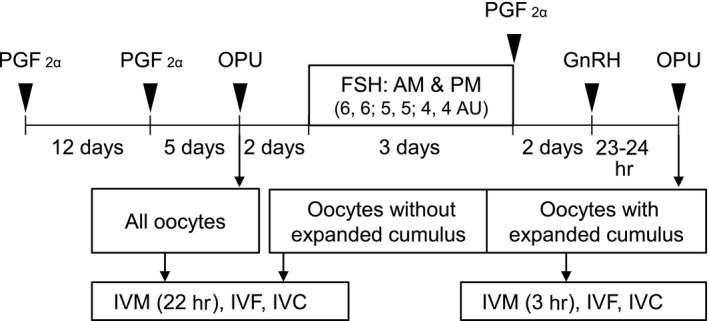
Schematic of ovum pick‐up (OPU) for *in vitro* embryo production. Nine Murrah buffaloes were used in this study. Buffaloes were injected with prostaglandin F_2α_ (PGF_2α_) with a 12‐day interval for synchronization. Five days after the second PGF_2α_ injection, buffaloes were subjected to OPU without superstimulation (control), and the oocytes collected were subjected to in vitro maturation (IVM, 22 hr), in vitro fertilization (IVF, 6 hr), and in vitro culture (IVC, 6 days). Two days after the first OPU, buffaloes were subjected to a follicle‐stimulating hormone (FSH) treatment that consisted of twice‐daily intramuscular injections for 3 days with a decreasing dose of FSH (6, 6, 5, 5, 4, and 4 AU per injection in the morning and afternoon for a total of 30 AU). At the last FSH injection, PGF_2α_ was also administered, and, after 2 days, gonadotropin‐releasing hormone (GnRH) was injected. The second OPU with superstimulation (superstimulated group) was conducted 23‐24 hr after the GnRH injection. Collected oocytes with expanded cumulus cells were defined as in vivo‐matured oocytes, and subjected to IVM (3 hr), IVF (6 hr), and IVC (6 days). Other collected oocytes were allocated as in vitro‐matured oocytes, and subjected to IVM, IVF, and IVC, similar to the non‐stimulated control

A schematic of the OPU schedule for in vitro embryo production is shown in Figure 1. Nine buffaloes were divided in three groups, and three cows in each group were simultaneously treated. The estrous cycles of three buffaloes were synchronized with prostaglandin F_2α_ (PGF_2α_; 25 mg; Lutalyse, dinoprost tromethamine, Pharmacia & Upjohn Co., MI, USA) injections at a 12‐day interval. Five days after the second PGF_2α_ injection, OPU using an ultrasound imaging device (HS‐2100; Honda Electronics, Aichi, Japan) equipped with a 9.0‐MHz long‐handled micro‐convex probe (HCV‐4710MV; Honda Electronics) was performed (control). The number of follicles in ovaries was counted, and follicles were classified by their diameter (small: <3 mm, middle: 3–8 mm, and large: >8 mm). Follicles were aspirated using a single‐lumen needle (17 gauge, length of 490 mm; Misawa Medical, Ibaraki, Japan) connected to a 50‐mL tube (Falcon 2070; Becton Dickinson, Franklin Lakes, NJ, USA) via a silicone tube (length of 100 cm, internal diameter of 1 mm). The collection tube was warmed at 37°C in a portable incubator (FV‐5; Fujihira Industry, Tokyo, Japan) and connected by a silicone tube to a vacuum pump (MODEL 4; Fujihira Industry). Two days after the first OPU, buffalo cows were subjected to the FSH treatment, which consisted of twice‐daily intramuscular injections for 3 days with a decreasing dose of FSH (6, 6, 5, 5, 4, and 4 AU per injection in the morning and afternoon for a total of 30 AU). At the last FSH injection, 25 mg PGF_2α_ was intramuscularly administered to induce luteolysis. Two days after the PGF_2α_ injection, GnRH (100 μg; Cystorelin, Merial Ltd., GA, USA) was intramuscularly injected to induce a LH surge, and we conducted the second OPU (superstimulated group) 23–24 hr after the GnRH injection because ovulation occurs 26–29 hr after the LH surge (average 27.7 hr) in buffalo species (Kanai & Shimizu, 1986). Regarding oocyte aspiration, we used a vacuum pressure of 55 mmHg as the control, and a higher vacuum pressure (70–80 mmHg) for the superstimulated group because of the previously reported presence of sticky cumulus cells (Matoba et al., 2014).

### 2.4 In vitro embryo production

Collected oocytes were washed using Dulbecco's phosphate‐buffered saline containing 1% newborn calf serum (NBCS) in a dish equipped with a filter (CCA200; Fujihira Industry), and their morphologies were evaluated under a stereomicroscope. Oocytes from three buffaloes in the same group were pooled and cultured in a 50‐μl droplet (2–12 oocytes/droplet) of IVM medium covered with paraffin oil in a 60‐mm plastic dish at 38.5°C under 5% CO_2_ in humidified air. After collection and classification, oocytes were subjected to several types of in vitro production protocols (Table S1). We used IVM medium consisting of HEPES‐buffered TCM‐199 supplemented with 0.2 mmol/L sodium pyruvate, 20 μg/mL FSH, 1 μg/mL estradiol valerate, 10 ng/mL epidermal growth factor, 50 μg/mL gentamicin sulfate, and 10% NBCS (TCM‐199 with NBCS) or 3 mg/mL fatty acid–free bovine serum albumin (TCM‐199 with BSA), or a commercially available cattle IVM medium (IVMD101; Research Institute for the Functional Peptides, Yamagata, Japan) (Table S1). After IVM, oocytes were co‐incubated with frozen‐thawed motile sperm (5.0 × 10^6^ cells/mL) from a buffalo bull in a 100‐μl droplet (2–12 oocytes/droplet) of IVF medium (IVF100; Research Institute for the Functional Peptides, Yamagata, Japan) covered with paraffin oil in a 35‐mm plastic dish at 38.5°C for 6 hr under 5% CO_2_ in humidified air. Presumptive zygotes were then co‐cultured with cumulus cells in a 30‐μl droplet (2–12 presumptive zygotes/droplet) of in vitro culture (IVC) medium covered with paraffin oil in a 35‐mm plastic dish at 38.5°C for 6 days under 5% CO_2_, 5% O_2_, and 90% N_2_ in humidified air. IVC medium consisted of modified synthetic oviduct fluid (mSOF) containing 1 mmol/L glutamine, 12 essential amino acids for basal medium Eagle, seven non‐essential amino acids for minimum essential medium, 10 μg/mL insulin, 5 mmol/L glycine, 5 mmol/L taurine, 1 mmol/L glucose, and 3 mg/mL fatty acid–free BSA, as previously described (Takahashi, Hishinuma, Matsui, Tanaka, & Kanagawa, 1996; Takahashi & Kanagawa, 1998). In some sessions, presumptive zygotes were transferred to IVC medium containing 5% NBCS instead of 3 mg/ml fatty acid–free BSA from days 3–6 of IVC (Atabay, Saturno, Atabay, & Maylem, 2017) (Table S1).

### 2.5 Experimental design

The numbers of follicles and oocytes collected by OPU from buffaloes were compared between the control and superstimulated groups. The morphologies of oocytes were evaluated by the cumulus investment conditions as denuded, partially denuded, monolayer, multilayer, and expanded. When oocytes in the superstimulated group had expanded cumulus investments, they were defined as in vivo‐matured and cultured for 3 hr (2–7 oocytes/droplet) in IVM medium. The in vivo‐matured oocytes of cattle collected by OPU 25–26 hr after the GnRH injection were cultured for 3 hr (Matoba et al., 2014). The remaining oocytes were cultured for 22 hr in droplets (5–12 oocytes/droplet) separated from in vivo‐matured oocytes. After IVF, cleavage and blastocyst rates were assessed on day 2 (approximately 42 hr) and day 6 (approximately 138 hr) of IVC, respectively. The percentage of transferable embryos was calculated based on the numbers of blastocysts and compacted morulae. In the first session of the control group, the concentration of CO_2_ gas was unstable due to a failure in the incubator, and we were unable to produce any blastocysts. Therefore, data were discarded, and we performed in vitro embryo production using oocytes (*n* = 60, 2 replicates) collected from slaughterhouse‐derived ovaries as the control without superstimulation (Table S1). These oocytes were cultured for IVM (15 oocytes/droplets) and IVC (15 oocytes/droplets) (Table S1). In this study, we combined the data of embryo development obtained from different culture systems because the development rates to blastocysts were similar regardless of the culture conditions employed in the present experiment (Table S2).

### 2.6 Statistical analysis

All statistical analyses were performed using software (StatView 4.51; Abacus Concepts, Inc., Calabasas, CA, USA). Data on the number of follicles at OPU and the number of oocytes collected by OPU were analyzed by the paired *t* test between the control and superstimulated groups. The percentages of cleavage on day 2 and blastocysts on day 6 were analyzed by the Student's *t* test between the control and superstimulated groups (in vitro‐matured).

## 3 RESULTS

At the OPU (5 days after PGF_2α_ injection) in the control group, one out of nine cows had a small CL (approximately 6 mm in diameter); however, others did not have CLs and were estimated at 1–3 days after estrus by ultrasonography. As shown in Table 1, the number of follicles smaller than 3 mm in diameter was higher in the control group (*p *<* *0.05), whereas the number of follicles larger than 8 mm in diameter was higher in the superstimulated group (*p *<* *0.05). The total number of follicles was higher in the superstimulated group than in the control group (*p *<* *0.05).

**Table 1:** Number of follicles of different sizes at ovum pick‐up in river buffaloes without (control) and with superstimulation (superstimulated)

Groups	No. of buffaloes	Number of follicles at OPU			
		<3 mm	3–8 mm	>8 mm	Total
Control	9	7.1 ± 2.7^a^	0.9 ± 0.8	1.0 ± 0.5	9.0 ± 2.7
Superstimulated	9	4.6 ± 2.4	1.3 ± 1.1	6.7 ± 4.2^a^	12.3 ± 4.2^a^
*p* values		0.049	0.500	0.004	0.044

Values are means ± *SD*.

a Asterisks indicate significant differences between groups (*p *<* *0.05).

As shown in Table 2, the total number of collected oocytes was similar regardless of superstimulation. The number of oocytes covered by multi‐layered cumulus cells was slightly higher in the superstimulated group than in the control group (*p *=* *0.05).

**Table 2:** Number of oocytes classified by cumulus investment collected by ovum pick‐up in river buffaloes without (control) and with superstimulation (superstimulated)

Groups	No. of buffaloes	Number of collected oocytes						
		Zona	Denuded	Partially	Monolayer	Multilayer	Expanded	Total
Control	9	0.0 ± 0.0	0.6 ± 1.0	1.7 ± 1.4	1.2 ± 1.5	0.2 ± 0.4	1.1 ± 1.1	4.8 ± 2.0
Superstimulated	9	0.2 ± 0.4	0.4 ± 0.7	0.7 ± 0.7	1.8 ± 1.2	0.8 ± 1.0	1.2 ± 1.5	5.1 ± 2.1
*p* values		0.169	0.824	0.122	0.401	0.051	0.834	0.688

Values are means ± *SD*.

Zona: Zona pellucida without an ooplasm. Denuded: Oocytes denuded from cumulus cells. Partially: Oocytes with partially attached cumulus cells. Monolayer: Oocytes covered by a monolayer of cumulus cells. Multilayer: Oocytes covered by multiple layers of cumulus cells. Expanded: Oocytes covered by expanded cumulus cells.

As shown in Table 3, no in vivo‐matured oocytes in the superstimulated group cleaved and developed to blastocysts. However, the percentages of cleavage and blastocysts from in vitro‐matured oocytes were higher in the superstimulated group than in the control group (*p *<* *0.05).

**Table 3:** Developmental competent oocytes from river buffalos without (control) or with superstimulation (Superstimulated)

Groups		No. of oocytes (replicates)	Percentages (ranges) of	
			Cleavage on day 2	Blastocysts on day 6
Control		91 (4)	27.9 ± 5.9^b^ (23.3‐35.7)	9.1 ± 3.3^b^ (5.8‐13.3)
Superstimulated	In vivo mature	11 (3)	0.0 (0.0)	0.0 (0.0)
	In vitro mature	33 (3)	61.8 ± 19.2^a^ (41.6–80.0)	21.6 ± 7.3^a^ (16.7–30.0)

Values are means ± *SD*.

_a,b_Different superscripts indicate significant differences within a column (*p *<* *0.05).

The control group includes two replicates of OPU‐IVF using oocytes collected from slaughterhouse‐derived ovaries. The superstimulated group includes three replicates of OPU‐IVF after superstimulation. In each replicate of OPU‐IVF, oocytes collected from three buffaloes were pooled.

## 4 DISCUSSION

In this study, we collected a larger number of oocytes covered by multi‐layered cumulus cells from the superstimulated group than from the control group (*p *=* *0.05). Sugimura et al. (2017) recently showed that the transcript levels of genes related to cell movement and migration were lower in granulosa cells derived from cattle treated with FSH before OPU than in those derived from cattle without a FSH treatment, which may prevent the disruption of the cell‐to‐cell connection in cattle. We speculate that the FSH treatment before OPU in buffaloes in this study also prevented the disruption of the connection between oocytes, cumulus cells, and each cumulus cell. Therefore, we were able to collect oocytes surrounded by multi‐layered cumulus cells in the superstimulated group

Moreover, the cleavage and blastocyst rates of the in vitro‐matured oocytes collected were higher in the superstimulated group than in the control group. The development rate to blastocysts was also higher than that in our previous study using OPU‐IVF without superstimulation (13.0%) (Aquino et al., 2013). Stojkovic et al. (2001) reported that mitochondrial distribution and adenosine triphosphate (ATP) contents in cattle oocytes correlated with the attachment of cumulus cells. Warriach and Chohan (2004) demonstrated that the nuclear maturation rates of buffalo oocytes with three or more cumulus cell layers (64.5%) and one or two cumulus cell layers (51.4%) were higher than oocytes with partial or no cumulus cells (8.6%). Furthermore, a co‐culture with cumulus cells increased nuclear maturation rates in oocytes with partial or no cumulus cells to 34.5% (Warriach & Chohan, 2004). These findings suggest that the superstimulation protocol promotes cell‐to‐cell connections between oocytes and cumulus cells, and increases mitochondrial reorganization and ATP production, resulting in the greater developmental competence of buffalo oocytes. In future studies, we need to examine mitochondrial function, ATP content in oocytes, and the function of granulosa (cumulus) cells after the superovulatory treatment.

In this study, the number of in vivo‐matured oocytes was small in the superstimulated group, and we were unable to produce blastocysts from these oocytes. One potential reason for the poor collection of in vivo‐matured oocytes was the lower vacuum pressure (70–80 mmHg) than that used for the collection of bovine in vivo‐matured oocytes (130 mmHg) (Matoba et al., 2014). Previous studies also used a lower pressure (40–100 mmHg) for the collection of in vivo‐matured oocytes from buffaloes (Presicce et al., 2002; Promdireg et al., 2005; Techakumphu et al., 2004) than from cattle. In our experience, cumulus cells surrounding buffalo oocytes were more easily removed from oocytes than those surrounding cattle oocytes. Therefore, we used a lower pressure than that for cattle, as reported previously (Matoba et al., 2014). In future studies, we need to establish a suitable vacuum pressure for collecting in vivo‐matured oocytes from buffaloes. Furthermore, we did not investigate the nuclear status of oocytes after the GnRH treatment. In addition, the morphology of cumulus investments judged as expanded collected by OPU was different from the expanded cumulus investments after IVM in buffalo oocytes (Jain et al., 2016), but similar to the cumulus investments of degenerating oocytes in cattle (De Wit & Kruip, 2001). In this study, we collected the oocytes having expanded cumulus investments not only in the superstimulated group but also in the control groups, suggesting that these oocytes are degenerating. Therefore, we need to confirm the appropriate timing of recovery oocyte after the GnRH treatment as well as the appropriate duration of the IVM culture for in vivo‐matured buffalo oocytes.

In conclusion, the FSH treatment followed by the GnRH injection in river buffaloes increased the number of large follicles at OPU and improved oocyte quality by enhancing the connection between oocyte and cumulus cells, thereby increasing embryo productivity by OPU‐IVF.
