# Supplementary material for: Effects of follicle‐stimulating hormone followed by gonadotropin‐releasing hormone on embryo production by ovum pick‐up and in vitro fertilization in the river buffalo (Bubalus bubalis)
Source: Anim Sci J. 2019 Mar 10;90(5):690–5. doi: 10.1111/asj.13196 (PMC6593430; doi:10.1111/asj.13196)
Supplement: Supplementary file 1 [file ASJ-90-690-s001.docx]

**SUPPORTING INFORMATION**

Table S1. Protocols for *in vitro* maturation (IVM) and *in vitro* culture (IVC) in each session

| Groups | | Date of IVF | IVM | IVC (mSOF a.i. ^3)^) |
| --- | --- | --- | --- | --- |
| Control | | 2017/6/27 | IVMD101  (22 h) | Presumptive zygotes only |
|  |  | 2017/7/4 | IVMD101  (22 h) | Co-culture with cumulus |
|  |  | 2017/7/11 | TCM-199 with NBCS ^1)^  (22 h) | Co-culture with cumulus →5% NBCS from day 3 of IVC |
|  |  | 2017/7/16* | TCM-199 with NBCS  (22 h) | Co-culture with cumulus →5% NBCS from day 3 of IVC |
|  |  |  | TCM-199 with BSA ^2)^  (22 h) | Co-culture with cumulus →5% NBCS from day 3 of IVC |
| Superstimulated | *In vivo*-matured | 2017/7/4 | IVMD101  (3 h) | Co-culture with cumulus |
|  |  | 2017/7/11 | TCM-199 with NBCS  (3 h) | Co-culture with cumulus →5% NBCS from day 3 of IVC |
|  |  | 2017/7/18 | TCM-199 with NBCS  (3 h) | Co-culture with cumulus →5% NBCS from day 3 of IVC |
|  | *In vitro*-matured | 2017/7/5 | IVMD101  (22 h) | Co-culture with cumulus |
|  |  | 2017/7/12 | TCM-199 with NBCS  (22 h) | Co-culture with cumulus →5% NBCS from day 3 of IVC |
|  |  | 2017/7/19 | TCM-199 with NBCS  (22 h) | Co-culture with cumulus  →5% NBCS from day 3 of IVC |

We used two types of IVM media and three types of IVC systems, which were culturing presumptive zygotes only, co-cultures with cumulus cells, and co-cultures with cumulus cells followed by a change to medium containing 5% newborn calf serum (NBCS). The IVF protocol was the same in each session (6 h in IVF-100).

* Oocytes were collected from slaughterhouse-derived ovaries.

^1)^ HEPES-buffered TCM-199 supplemented with 0.2 mmol/L sodium pyruvate, 20 μg/mL FSH, 1 μg/mL estradiol valerate, 10 ng/mL epidermal growth factor, 50 μg/mL gentamicin sulfate, and 10% NBCS

^2)^ HEPES-buffered TCM-199 supplemented with 0.2 mmol/L sodium pyruvate, 20 μg/mL FSH, 1 μg/mL estradiol valerate, 10 ng/mL epidermal growth factor, 50 μg/mL gentamicin sulfate, and 3 mg/mL fatty acid–free BSA

^3)^ Modified synthetic oviduct fluid containing 1 mmol/L glutamine,12 essential amino acids for basal medium Eagle, 7 non-essential amino acids for minimum essential medium, 10 μg/mL insulin, 5 mmol/L glycine, 5　mmol/L taurine, 1 mmol/L glucose, and 3 mg/mL fatty acid–free BSA or 5% NBCS.

Table S2. Developmental competence of oocytes from river buffaloes subjected to serum-free *in vitro* culture (IVC) or cultured in medium containing 5% serum from day 3.

| Groups | No. of oocytes (replicate) | Percentage (range) of blastocysts on day 6 |
| --- | --- | --- |
| Serum-free | 24 (2) | 16.7 (7.1-30.0) |
| 5% serum | 100 (4) | 12. 0 (5.9-18.2) |
| *P* value | | 0.540 |

In the serum-free culture group, presumptive zygotes were cultured for 6 days in IVC medium consisting of modified synthetic oviduct fluid containing 1 mmol/L glutamine,12 essential amino acids for basal medium Eagle, 7 non-essential amino acids for minimum essential medium, 10 μg/mL insulin, 5 mmol/L glycine, 5 mmol/L taurine, 1 mmol/L glucose, and 3 mg/mL fatty acid–free BSA. In the 5% serum group, presumptive zygotes were transferred to IVC medium containing 5% NBCS instead of 3 mg/mL fatty acid–free BSA from day 3 of IVC.
